# Identifying Alpha-1 Antitrypsin Deficiency Based on Computed Tomography Evidence of Emphysema

**DOI:** 10.7759/cureus.3971

**Published:** 2019-01-28

**Authors:** Jeffrey A Miskoff, Bilal Khan, Moiuz Chaudhri, Hai Phan, Michael P Carson

**Affiliations:** 1 Internal Medicine, Jersey Shore University Medical Center, Neptune City, USA; 2 Internal Medicine, Bayonne Medical Center, Bayonne, USA; 3 Internal Medicine, JFK Medical Center, Atlantis, USA

**Keywords:** alpha 1 antitrypsin deficiency, diagnostic imaging, emphysema, genotype, lung diseases, chronic obstructive pulmonary disease, bronchitis, smoking, respiratory, pulmonary function test

## Abstract

Introduction

Chronic obstructive pulmonary disease (COPD) is most commonly caused by smoking tobacco or cigarettes. However, alpha-1 antitrypsin deficiency (AATD) is the only genetic disorder known to cause COPD and these patients often present with emphysema earlier in life and with more severe disease. Additionally, AATD patients are often misdiagnosed with other lung disorders, and the diagnosis is often delayed for up to a decade. Furthermore, several clinicians may see the patient before genetic testing is performed and an official diagnosis is made. We hypothesized that patients with radiographic emphysema on computed tomography (CT) scan of the chest would represent an enriched population of patients with a higher prevalence of alpha-1 antitrypsin (AAT) carrier or heterozygous state.

Methods

We evaluated 250 in-patients with chest computed tomography (CT) findings of emphysema, and per clinical guidelines, all were tested for AAT with Alphakit finger stick blood collection kits. Sampling 250 patients provided power to detect a carrier prevalence of 20% +/- 1.0%.

Results

A total of 250 patients were recruited of which 53% were male, 91% Caucasian, 7% African American, and 16% active smokers. They smoked an average of 39 packs per year. The prevalence of carrier status (Pi*MS or Pi*MZ) was 6.8% (95% CI (4%, 11%)). The mean forced expiratory volume in one second (FEV-1) was 53%, predicted among Pi*MM patients (n=126) and not significantly different from the Pi*MS group (50%, n=13). 69% of Pi*MM were diagnosed with asthma or COPD, vs. 79% of Pi*MS (n=14) and 100% Pi*MZ (n=3), but the difference was not significant (p=0.4).

Conclusion

In the population studied, compared to a cohort of patients with abnormal pulmonary function tests (PFTs), radiographically evident emphysema did not identify patients at higher risk of being heterozygous or homozygous for AAT deficiency.

## Introduction

Chronic obstructive pulmonary disease (COPD), which includes chronic bronchitis and emphysema, is characterized by air trapping, progressive airflow limitations, and chronic inflammatory response in the lung. COPD is a significant cause of disability and the third leading cause of death in the United States [1]. More than 11 million people are currently diagnosed with COPD, and it is estimated that up to 24 million may have the disease [1]. In addition to smoking, COPD can result from a congenital deficiency in the protease inhibitor (Pi) of the proteolytic enzyme elastase known as alpha-1 antitrypsin, belonging to the class of serine protease inhibitors (serpins). Abnormalities of serine inhibitors are also associated with neurodegenerative diseases, coagulation abnormalities, and angioedema [2-3]. Evidence suggests that emphysema in alpha-1 antitrypsin deficiency (AATD) is related to an imbalance between the proteolytic enzyme, neutrophil elastase, in the lung and the protecting enzyme, which is made in the liver [3]. AATD results from a mutation in the SERPINA1 gene (long arm of chromosome 14) located at 14q32.1, which produces the AAT protein in the liver [4]. Normally, this protein gets released in the blood and is responsible for protecting lung tissue from the neutrophil elastase. In this condition, the mutation of the SERPINA1 gene leads to the production of an abnormal protein that gets trapped in the liver, resulting in low serum levels of AAT protein leading to not being able to protect against the degradation caused by neutrophil elastase. Furthermore, the accumulation of abnormal protein in the liver over decades may increase the risk of cirrhosis and other liver malignancies such as hepatoma [5-7].

Alpha-1 antitrypsin deficiency is a congenital condition, inherited in autosomal co-dominant transmission [8]. The normal AAT genotype is Pi*MM (Pi = protease inhibitor), the most common severe deficiency allele is the Z allele, and individuals with two defective copies of Z present with AAT plasma levels that are 15% of the normal (≥100 mg/dL). In contrast, the S allele represents moderate disease, therefore, individuals with two defective copies of S present with AAT plasma levels that are 60% of the normal [4,8-9]. Various estimates suggest that in the United States, the phenotypic prevalence is one in 17 for Pi*MS, one in 4775 for Pi*ZZ, one in 1124 for Pi*SZ, one in 1058 for Pi*SS, and one in 36 for Pi*MZ [10]. Data suggest that severe deficiency is associated with a single base pair substitution of glutamate to lysine at position 342 and glutamate to valine mutation occurring at position 264 [9,11]. Currently, up to 25 million Americans are estimated to have an abnormal gene for the production and release of AAT [12]. Data suggest that up to 100,000 may have a clinically significant AAT deficiency in the United States [10,13]. Patients with one normal (Pi*M) and one defective gene (Pi*S or Pi*Z) are carriers who may pass the defective gene on to their children. Historically, the risk for emphysema among non-smoking heterozygotes (Pi*MS/Pi*MZ) is not extensively studied, but the most recent data suggest a history of ever smoking tobacco increases the risk of airflow obstruction in the MZ heterozygotes [12].

Although AATD is a unique condition, it can be challenging to differentiate AATD-related COPD from other etiologies due to shared signs and symptoms [3,14]. A study of St. Louis area residents conducted direct population screening from 20,000 blood donors, seven Pi*Z AAT-deficient persons were identified and confirmed, which is more than twice the number predicted from previous estimates of the Z allele frequency in the St. Louis area [15]. A poll of 304 individuals with severe AAT deficiency indicated an average delay of 7.2 years between the onset of symptoms and the diagnosis. Two subsequent surveys demonstrated that the delay between the first symptoms and recognition of AAT deficiency had not decreased, indicating that under-recognition persists despite evidence-based guidelines for the diagnosis of AAT deficiency [13,16]. 

White Pi*MZ patients are more likely to have radiographic evidence of emphysema than non-carriers, yet, currently, no guidelines specifically recommending AAT testing for patients with evidence of emphysema on chest CT [12-13]. For this reason, we hypothesized that evidence of COPD noted on chest CT scans may be a marker that would identify patients with a higher prevalence of AAT heterozygous or homozygous states and thus be a tool that could improve the rate at which these patients are identified. Such findings could provide evidence to modify screening AAT criteria with the addition of emphysema on chest CT.

## Materials and methods

Inclusion criteria for this prospective, cross-sectional cohort study were: admission to Jersey Shore University Medical Center (JSUMC), radiological evidence of COPD/emphysema finding on chest CT, as determined by the radiology report, along with a pattern of emphysema. In line with current consensus guidelines, the clinical team offered AAT testing to patients with these findings and the risks/benefits were reviewed before testing [9]. AAT testing was conducted using the AlphaKit test kit supplied by Grifols (Los Angeles CA, USA/University of Florida). The test involves a finger-stick to collect three drops of blood sample onto the test kit. The properly labeled test kit was placed in a secure envelope and sent to the AAT laboratory at the University of Florida. Each patient was provided with a brochure “What is Alpha-1 Antitrypsin Deficiency? Should I be tested?" All test results were sent to the pulmonary attending office for further review.

Patient demographics for this study included age, gender, race, current medications, history of smoking, including the number of pack-years, and past medical history, including COPD/asthma. Furthermore, additional metrics, such as another underlying lung disease, liver disease, lung cancer, most recent pulmonary function tests, and location/type of emphysema/COPD on chest CT, were included.

Statistical analysis

Historically, the AAT carrier rate in patients with fixed airflow obstruction on pulmonary function testing is 10.8% (Pi*MS or Pi*MZ genotype), the prevalence of the Pi*SZ/Pi*ZZ genotype was 0.63%, and 8.8% are estimated to have phenotypic AAT (Pi*MS, Pi*MZ, Pi*SS, Pi*SZ, and Pi*ZZ) [10,17]. We hypothesized the following: 1) the presence of objective anatomic evidence of COPD on computed tomography (CT) of the chest/lungs would identify a population with greater disease severity as compared to those who only had evidence of airflow obstruction; 2) that these patients, theoretically with more severe lung disease, would be more likely to have an abnormal genotype; and 3) the carrier status prevalence in the population with CT evidence of COPD would be 50% greater than the historical cohort with airflow obstruction (estimated absolute prevalence of 17%). Reviewing 217 charts would give us a 95% chance to detect that rate with a +/- 0.8% absolute error rate. As 250 testing kits were provided by the manufacturer, we expanded the sample size to 250. The test results were classified based on genotype: Pi*MM (normal), Pi*MS/Pi*MZ (heterozygote, carrier), and Pi*ZZ (homozygote). The data from these groups were characterized using descriptive statistics, and 95% confidence interval was calculated regarding the rate of heterozygosity. STATA 10 (College Station TX, USA) was used for analysis. For nonparametric results, the Kruskal-Wallis test was used to compare means between three categories, the Wilcoxon rank-sum between two categories, and Fisher’s exact test to compare categorical variables when a cell value was <5. Meridian Health IRB approval was obtained (Study# 201307181J).

## Results

Samples were obtained from a total of 250 patients between December 2012 and May 2014. Demographics (Table 1; using the Wilcoxon rank-sum): 53.6% were male, the median age for the entire population was 73 years, 91% were self-classified as white, and 7.25% African American. The average pack-year smoking history was 39, and 14.8% of the population were active smokers.

**Table 1:** Patient Demographics Stratified by Alpha-1 Antitrypsin Genotype Pi*MM=Normal. Pi*MS=carrier of deficiency allele S. Pi*MZ=carrier of deficiency allele Z. IQR: Interquartile Ratio

Variable	Pi*MM (Row %)	Pi*MS (Row %)	Pi*MZ (Row %)	Total (Column %)
Gender, n (%)				
Male	124 (92%)	9 (7%)	1 (1%)	134 (54%)
Female	109 (94%)	5 (4%)	2 (2%)	116 (46%)
Age, Median Years [IQR]	73 [+/- 11]	73 [+/-9]	78 [+/-3]	73 [+/-11]
Race, n (%)				
White	212 (94%)	12 (5%)	2 (1%)	226 (90.5%)
African-American	16 (89%)	1 (5.5%)	1 (5.5%)	18 (7.0%)
Asian	0 (0%)	1 (100%)	0 (0%)	1 (0.5%)
American Indian	1 (100%)	0 (0%)	0 (0%)	1 (0.5%)
Other	4 (100%)	0 ( 0%)	0 (0%)	4 ( 1.5%)
Active smoking				
Yes	37 (100%)	0 ( 0%)	0 (0%)	37 (15%)
No	196 (92%)	14 (7%)	3 (1%)	213 ( 85%)
Pack Year Smoking	39 [+/-29]	37 [+/-29]	50 [+/-10]	39 [+/-28]

Primary outcome

Of the total 250 patients, 233 patients were Pi*MM, 14 were Pi*MS, three Pi*MZ, none PI*ZZ. The prevalence of carrier status (Pi*MS or Pi*MZ) was 6.8% (95% CI (4%,11%)), similar to historical rates and lower than our hypothesized rate of 17%.

Table 2 (using Fisher’s exact test) lists comorbidities stratified by genotype: 69% of Pi*MM were diagnosed with COPD vs. 79% of Pi*MS (n=14) and 100% Pi*MZ (n=3), but the difference was not significant (Fisher’s exact test p=0.52). None of the Pi*MZ patients has a liver disease. The rates of lung cancer were not different, the mean ejection fraction stratified by phenotype was 55% in all three groups, and the only patient with a history of hepatic cirrhosis was Pi*MM, although this was not a primary endpoint of this study and any observations here are likely limited by sample size.

**Table 2:** Past Medical History as Recorded in the Medical Record Stratified by Genotype COPD: Chronic Obstructive Pulmonary Disease. CAD: Coronary Artery Disease. CABG: Coronary Artery Bypass Graft. CKD: Chronic Kidney Disease. CHF: Congestive Heart Failure

Past medical history	Pi*MM (Column %)	Pi*MS (Column %)	Pi*MZ (Column %)	Total
COPD				
Yes	156 (67%)	11 (79%)	3 (100%)	170 (68%)
No	77 (33%)	3 (21%)	0 (0%)	80 (32%)
Asthma				
Yes	19 (100%)	0 (0%)	0 (0%)	19 (7.6%)
No	214 (93%)	14 (6%)	3 (1%)	231 (92.4%)
Hepatic Cirrhosis				
Yes	1 (0.5%)	0 (0%)	0 (0%)	1 (1%)
No	232 (99.5%)	14 (100%)	3 (100%)	249 (99%)
Lung cancer				
Yes	29 (12%)	3 (21%)	1 (33%)	33 (13%)
No	204 (88%)	11 (79%)	2 (67%)	217 (87%)
Systolic CHF				
Yes	24 (10%)	4 (29%)	0 (0%)	28 (11%)
No	209 (90%)	10 (71%)	3 (100%)	222 (89%)
Hypertension				
Yes	152 (65%)	5 (36%)	1 (33%)	158 (63%)
No	81 (35%)	9 (64%)	2 (67%)	92 (37%)
CAD				
Yes	88 (38%)	5 (64%)	0 (0%)	93 (37.20%)
No	145 (62%)	9 (36%)	3 (100%)	157 (62.80%)
CABG				
Yes	25 (11%)	1 (7%)	0 (0%)	26 (10%)
No	208 (89%)	13 (93%)	3 (100%)	224 (90%)
CKD				
Yes	31 (13%)	1 (7%)	1 (33%)	33 (13%)
No	202 (87%)	13 (93%)	2 (67%)	217 (87%)
Diabetes Mellitus				
Yes	49 (21%)	4 (29%)	1 (33%)	54 (22%)
No	184 (79%)	10 (71.43%)	2 (67%)	196 (78%)

Table 3 (using the Kruskal-Wallis test) describes the results of the pulmonary function test and chest CT results. In our cohort, 111 of the 250 patients did not have PFT results in the hospital system or office records of the investigators and thus were not available for analysis as part of this study. Mean FEV-1% was 53% predicted among Pi*MM patients (n=126) and not significantly different from the Pi*MS/Pi*MZ group (50%, n=13). None of the Pi*MS/Pi*MZ carriers has basilar distribution type of emphysema on chest CT. No patients had the ZZ genotype.

**Table 3:** Pulmonary Test Results Stratified by AAT Genotype AAT: Alpha-1 Antitrypsin. CT: Computed Tomography. DLCO: Diffusion of Carbon Monoxide. FEV-1: Forced Expiratory Volume in one second. FVC: Forced Vital Capacity. FEV-1%: Forced Expiratory Volume one second/Vital Capacity. PFT: Pulmonary Function Test. S.D. = Standard Deviation. * No significant differences in mean values stratified by phenotypic group.

Variable	Pi*MM (n=233)	MS (n=14)	MZ (n=3)	Total (n=250)
PFT				
FEV-1 (%+S.D.)*	53+24 (n=126)	50+25 (n= 10)	48+26 (n=3)	53+24 (n=139)
FEV-1/FVC (%)*	60+17 (n=126)	56+18 (n=10)	60+10 (n=3)	60+17 (n= 139)
DLCO (%)*	79+153 (n=82)	44+15 (n=5)	71 (n=1)	77+148 (n= 88)
Type Of Emphysema Observed On Chest CT	(Column %)	(Column %)	(Column %)	(Column %)
Diffuse	106 (45%)	6 (42%)	0	112 (45%)
Centrilobular	38 (16%)	4 (29%)	0	42 (17%)
Basal	14 (6%)	0	0	14 (6%)
Apical	68 (30%)	4 (29%)	3 (100%)	75 (30%)
Peripheral	6 (2.5%)	0	0	6 (2%)
Panacinar	1 (0.5%)	0	0	1 (0.4%)

## Discussion

Alpha-1 antitrypsin deficiency (AATD) is a clinically under-recognized inherited disorder, which primarily affects the lungs and liver. The severity of AATD depends on whether the patient is deficient in one or two copies of a defective allele(s). The mean forced expiratory volume in 1-second (FEV-1%) between our groups was not significantly different. The values in this group with COPD on chest CT averaged 60% (Table 3) vs. a prior study of patients with airflow obstruction on pulmonary function tests (PFT) who averaged 50%. It is logical that patients screened based on the presence of fixed airflow obstruction would have lower values than patients screened based on referral to a pulmonary consultation service for varied indications [17].

Although the prevalence of AAT carrier status varies geographically, estimates suggest that it affects about one in 1,500 to 3,500 individuals with European ancestry [10]. Historically, the rate of AAT carrier status was 10.9% in patients with COPD based on PFT results, so our theory was that patients with enough lung damage to be visualized on CT imaging would have a degree of disease severity similar to or more severe than those diagnosed via PFTs [17]. The sample size of our cohort was adequate to detect 20% carrier status prevalence; therefore, it is more likely the observed carrier rate of 6.8% in patients with radiographically documented emphysema was identified because the null hypothesis is true and emphysema CT scan is not a more sensitive tool.

Alpha-1 antitrypsin deficiency (AATD) screening recommendations have been simplified from their original 83-page executive summary published in 2003 to facilitate broad clinical application [9,18]. Therefore, in addition to patients with the onset of emphysema < age 45, emphysema without history of smoking, pulmonary function test decline more than expected for a non-smoker or smoker who stopped tobacco, predominantly basilar emphysema changes on the chest radiograph, a family history emphysema or/and liver disease, clinical findings or history of panniculitis, or clinical findings or history of unexplained chronic liver disease can be used to guide clinical suspicion [9,18].

Despite these guidelines, screening and diagnosing AATD remains a challenge. A study by Campos et al. stated that from 1968 to 2003, the mean age at which AAT was diagnosed increased and 30.8% of patients diagnosed were older than the age of 50 [13]. In addition, the average interval from symptoms to diagnosis was 8.3 + 6.9 years, 20% of patients require evaluations by four or more physicians to make the diagnosis, and fewer patients were diagnosed by the first or second physician they saw [13,16]. A follow-up study by Campos et al. showed that more than 50% of patients were diagnosed at the age older than 50 [19]. This suggests that the clinicians are testing older patients as recommended by the simplified guidelines. Our theory was that chest CTs, being more sensitive than plain radiography, could be an additional tool that would help reverse the trend of diagnoses being made later in life.

Although there are limits generalizing a study performed at a single institution, our sample size was large enough to detect high prevalence; however, chances of detecting a difference was improved by the fact that AAT is classically a disease of White Europeans and 90% of our population was classified as White. This negative study contributes to the growing body of knowledge, as it is the first to report AAT prevalence in a group with a specific set of chest CT findings [10]. While White Pi*MZ carriers in one study (n=261) were more likely to have emphysema on CT scans as compared to Pi*MM (11% vs. 8%), our data (n=250) does not support the reverse being true [12].

## Conclusions

In the context of the factors noted, we conclude that in the population studied, radiographically evident emphysema on chest CT, contrary to our hypothesis, does not identify patients at higher risk of being heterozygous or homozygous for AAT deficiency and, therefore, these data do not yet support a need to modify current screening recommendations. Also, the data should not be interpreted to imply that testing all patients with emphysema on chest CT be considered overtesting. In fact, current and older guidelines by the American Thoracic Society (ATS) supports testing all patients with “symptomatic emphysema” for AATD. Our patients almost universally have either dyspnea, cough, or wheeze and did have emphysema. The majority of patients with severe AATD are still not diagnosed and, therefore, more testing not less is still needed.

## References

[1] Minino AM, Murphy SL, Xu J, Kochanek KD (2011). Deaths: final data for 2008. Natl Vital Stat Rep.

[2] Carrell RW, Lomas DA (2002). Alpha1-antitrypsin deficiency — a model for conformational diseases. N Engl J Med.

[3] Stoller JK, Aboussouan LS (2012). A review of α1-antitrypsin deficiency. Am J Respir Crit Care Med.

[4] McGee D, Schwarz L, McClure R, Peterka L, Rouhani F, Brantly M, Strange C (2010). Is PiSS alpha-1 antitrypsin deficiency associated with disease?. Pulm Med.

[5] Alam S, Li Z, Janciauskiene S, Mahadeva R (2011). Oxidation of Z alpha1-antitrypsin by cigarette smoke induces polymerization: a novel mechanism of early-onset emphysema. Am J Respir Cell Mol Biol.

[6] O'Brien M, Pennycooke K, Carroll T (2015). The impact of smoke exposure on the clinical phenotype of alpha-1 antitrypsin deficiency in Ireland: exploiting a national registry to understand a rare disease. COPD.

[7] Silva D, Oliveira MJ, Guimaraes M, Lima R, Gomes S, Seixas S (2016). Alpha-1-antitrypsin (SERPINA1) mutation spectrum: three novel variants and haplotype characterization of rare deficiency alleles identified in Portugal. Respir Med.

[8] Craig TJ, Henao MP (2018). Advances in managing COPD related to alpha1 -antitrypsin deficiency: an under-recognized genetic disorder. Allergy.

[9] American Thoracic Society, European Respiratory Society (2003). American Thoracic Society/European Respiratory Society statement: standards for the diagnosis and management of individuals with alpha-1 antitrypsin deficiency. Am J Respir Crit Care Med.

[10] de Serres F, Blanco I, Fernandez-Bustillo E (2003). Genetic epidemiology of Alpha‐1 antitrypsin deficiency in North America and Australia/New Zealand: Australia, Canada, New Zealand and the United States of America. Clin Genet.

[11] Fregonese L, Stolk J (2008). Hereditary alpha-1-antitrypsin deficiency and its clinical consequences. Orphanet J Rare Dis.

[12] Foreman M, Wilson C, DeMeo D (2017). Genetic epidemiology of CI: alpha-1 antitrypsin PiMZ genotype is associated with chronic obstructive pulmonary disease in two racial groups. Ann Am Thorac Soc.

[13] Campos MA, Wanner A, Zhang G, Sandhaus RA (2005). Trends in the diagnosis of symptomatic patients with alpha1-antitrypsin deficiency between 1968 and 2003. Chest.

[14] Petrache I, Hajjar J, Campos M (2009). Safety and efficacy of alpha-1-antitrypsin augmentation therapy in the treatment of patients with alpha-1-antitrypsin deficiency. Biologics.

[15] Silverman E, Miletich J, Pierce J, Sherman LA, Endicott SK, Broze GJ Jr, Campbell EJ (1989). Alpha-1-antitrypsin deficiency. High prevalence in the St. Louis area determined by direct population screening. Am Rev Respir Dis.

[16] Stoller JK, Sandhaus RA, Turino G, Dickson R, Rodgers K, Strange C (2005). Delay in diagnosis of alpha1-antitrypsin deficiency: a continuing problem. Chest.

[17] Rahaghi F, Sandhaus R, Brantly M (2012). The prevalence of alpha-1 antitrypsin deficiency among patients found to have airflow obstruction. COPD.

[18] Sandhaus R, Turino G, Brantly M (2016). The diagnosis and management of alpha-1 antitrypsin deficiency in the adult. Chronic Obstr Pulm Dis.

[19] Campos MA, Alazemi S, Zhang G, Salathe M, Wanner A, Sandhaus RA, Baier H (2009). Clinical characteristics of subjects with symptoms of alpha1-antitrypsin deficiency older than 60 years. Chest.

